# Nurse forecasting in Europe (RN4CAST): Rationale, design and methodology

**DOI:** 10.1186/1472-6955-10-6

**Published:** 2011-04-18

**Authors:** Walter Sermeus, Linda H Aiken, Koen Van den Heede, Anne Marie Rafferty, Peter Griffiths, Maria Teresa Moreno-Casbas, Reinhard Busse, Rikard Lindqvist, Anne P Scott, Luk Bruyneel, Tomasz Brzostek, Juha Kinnunen, Maria Schubert, Lisette Schoonhoven, Dimitrios Zikos, RN4CAST consortium

**Affiliations:** 1 Center for Health Services and Nursing Research, Katholieke Universiteit Leuven, Kapucijnenvoer 35/4, 3000 Leuven, Belgium; 2 Center for Health Outcomes and Policy Research, University of Pennsylvania, 418 Curie Blvd. Claire M. Fagin Hall, 387R, Philadelphia, PA 19104-4217, USA; 3 Florence Nightingale School of Nursing & Midwifery, King's College London, James Clerk Maxwell Building, 57 Waterloo Road, London SE1 8WA, UK; 4 School of Health Sciences, University of Southampton, Building 67, Highfield Campus, Southampton 17 1BJ, UK; 5 National Spanish Research Unit, Instituto de Salud Carlos III. Ministry of Science and Innovation, C/Monforte de Lemos, 5. Pabellón 13, 28029 Madrid, Spain; 6 Department of Health Care Management, WHO Collaborating Centre for Health Systems Research and Management, Technische Universität Berlin, H 80, Strasse des 17. Juni 135, 10623 Berlin, Germany; 7 Department of Learning, Informatics, Management and Ethics, Karolinska Institutet, 171 77 Stockholm, Sweden; 8 School of Nursing, Dublin City University, Dublin 9, Ireland; 9 Department of Internal Diseases and Community Nursing, Jagiellonian University Medical College, Kopernika 25, 31-501 Krakow, Poland; 10 Department of Health Policy and Management, University of Eastern Finland, POB 1627, 70211 Kuopio, Finland; 11 Institute of Nursing Science, University of Basel, Bernoullistrasse 28, 4056 Basel, Switzerland; 12 Scientific Institute for Quality of Healthcare, UMC St Radboud, Postbus 9101, 114 IQ healthcare, 6500 HB Nijmegen, The Netherlands; 13 Laboratory of Health Informatics, Faculty of Nursing, National and Kapodistrian University of Athens, Papadiamantopoulou 123, 11527 Athens, Greece

## Abstract

**Background:**

Current human resources planning models in nursing are unreliable and ineffective as they consider volumes, but ignore effects on quality in patient care. The project RN4CAST aims innovative forecasting methods by addressing not only volumes, but quality of nursing staff as well as quality of patient care.

**Methods/Design:**

A multi-country, multilevel cross-sectional design is used to obtain important unmeasured factors in forecasting models including how features of hospital work environments impact on nurse recruitment, retention and patient outcomes. In each of the 12 participating European countries, at least 30 general acute hospitals were sampled. Data are gathered via four data sources (nurse, patient and organizational surveys and via routinely collected hospital discharge data). All staff nurses of a random selection of medical and surgical units (at least 2 per hospital) were surveyed. The nurse survey has the purpose to measure the experiences of nurses on their job (e.g. job satisfaction, burnout) as well as to allow the creation of aggregated hospital level measures of staffing and working conditions. The patient survey is organized in a sub-sample of countries and hospitals using a one-day census approach to measure the patient experiences with medical and nursing care. In addition to conducting a patient survey, hospital discharge abstract datasets will be used to calculate additional patient outcomes like in-hospital mortality and failure-to-rescue. Via the organizational survey, information about the organizational profile (e.g. bed size, types of technology available, teaching status) is collected to control the analyses for institutional differences.

This information will be linked via common identifiers and the relationships between different aspects of the nursing work environment and patient and nurse outcomes will be studied by using multilevel regression type analyses. These results will be used to simulate the impact of changing different aspects of the nursing work environment on quality of care and satisfaction of the nursing workforce.

**Discussion:**

RN4CAST is one of the largest nurse workforce studies ever conducted in Europe, will add to accuracy of forecasting models and generate new approaches to more effective management of nursing resources in Europe.

## Background

All countries, rich and poor, have numeric, skill, and geographic imbalances in their healthcare and nursing workforce [1] and are lacking an adequate nurse workforce to meet projected future requirements for care. This global nurse shortage is remarkable in light of the highly reported variability in nurse density (number of nurses per 1000 inhabitants) across countries. In Europe, for example, the highest (Ireland: 14.8) nurse density is nearly 4 times higher than the lowest nurse density (Greece: 3.8) [2]. The observed variation in nurse density seems, apparently, to be independent from the reported shortages of nursing personnel across European countries. This can, possibly, be explained by the definition and measurement of shortages. Nursing shortages on the country level are mostly viewed in relation to that country's own historical staffing levels and resources [3]. Driven by ageing populations, demand for healthcare and for nurses will continue to grow, whilst the supply of available nurses will drop [3,4]. Therefore, it is expected that the shortages will accelerate in the coming decade and will be more serious than the cyclical shortages of the past [3]. This nursing shortage will ultimately constrain health system reform and innovation, and contribute to escalating costs [5]. Recent analyses of global human resources for health conclude that all countries can accelerate health gains through more strategic investments in and management of their nursing workforces [6,7]. However, nursing workforce planning and forecasting efforts have a poor record both of accurately predicting future nursing workforce needs and of informing policy interventions that avoid cyclical shortages. The multiplicity of inputs and consequences of societal, health systems, and professional trends, makes the determination of the optimal number of nurses for any given country very complex. The conclusion of a review of current forecasting methods was that they all show serious shortcomings in terms of comprehensiveness and accuracy of forecasts [8]. The most simple approaches use only the ratio of healthcare workers for their predictions [9]. Other country-specific forecasts of the need for nursing personnel generally take into account demand as well as supply factors based on historically established staffing levels, resources and estimates of demand for health services.

To our knowledge none of these models take into account the dynamics between nurse-to-patient ratios, skill mix, nurses' education level, the nursing work environment on one hand and nurse outcomes (nurse retention, job satisfaction, burnout) and patient outcomes on the other hand. Evidence, nonetheless, confirms that effective nursing workforce strategies enhance the performance of health care organizations and health systems [10-16]. It was shown, for example, that after the implementation of mandated minimum nurse-to-patient ratio's in California the nurse staffing levels in hospitals increased substantially. Aiken et al. [12] illustrated that these lower workloads (i.e. Californian nurses are caring on average for one patient less in comparison to nurses in other states) were associated with lower patient mortality, as well as burnout, job dissatisfaction and better nurse-reported perceived quality of care.

The main aim of the RN4CAST-study is to expand and refine typical forecasting models with factors that take into account how features of work environments and qualifications of the nurse workforce impact on nurse retention, burnout among nurses and patient outcomes. The RN4CAST-study aims to simulate scenarios to illustrate what happens to the quality of patient care and nursing outcomes when different aspects of the nursing workforce (nurse-to-patient ratio, nurse education, nurse skill mix, nursing work environment) are changed.

## Methods/Design

This 3-year project involves two major phases. The first phase is focused on instrument development and data gathering (January 2009-June 2010), whereas the second study phase is focused on data analysis and policy synthesis (July 2010-December 2011).

A common international protocol was written to standardize data collection procedures, instruments, and training of staff and to enable comparability of measures across sites and to facilitate cross-country analyses. At the same time, the study protocol integrates flexibility to allow for differences in the health system structures and nature of the nursing workforces in each country. All differences included in the national study protocols had to be reported by each collaborating team to and approved by the coordinating center.

This study makes use of a cross-sectional multilevel design with data collected at the hospital, nursing unit, individual nurse and patient level via four different data sources. A first data source is a survey of the general hospital management about general hospital-wide characteristics like bed size, teaching status and technology level. A second data source is a survey of nurses. Nurses serve as informants about organizational characteristics (e.g. nursing work environment) situated at the nursing unit or hospital level. In addition nurses are also surveyed about individual nurse outcomes like job satisfaction, intention to leave the hospital and burnout. A third data source is routinely collected administrative databases, used to derive patient level data on mortality and other patient outcomes. From the start, it was anticipated that in some countries the availability of routinely collected patient level data is limited, of poor quality or the process to acquire data access could be time consuming hampering to meet the project deadlines. Therefore, in a sub-sample of countries and hospitals a patient survey was conducted to ensure that patient level data can be included in the analysis within the time limits of the project. The different data sources can be linked on the level of the nursing unit/hospital by means of common identifiers.

### Setting and sample

Twelve European countries (Belgium, England, Finland, Germany, Greece, Ireland, Norway, Poland, Spain, Sweden, Switzerland, and The Netherlands) were selected on the basis of research expertise, availability of patient discharge data from hospitals, geographic distribution, and duration of membership in the European Union. There are many similarities in healthcare in these selected countries, but also some striking differences, particularly in the size and structure of their healthcare workforces, the percentage of Gross Domestic Product spent on health care and the average length of acute hospital care stays [17]. The themes of similarity and difference within Europe create unique opportunities to study nursing workforce issues while learning common lessons across countries.

The setting for the RN4CAST-study focused on general acute hospitals (with at least 100 beds) that either have mixed age clienteles or treat adults only. This setting was chosen since general acute hospitals are the largest employers of nurses [17] and thus exert major influence on demand for nurses in most countries. In addition general acute hospitals represent, the largest share of national health expenditures [17] and are the sites of the largest proportion of medical errors leading to serious injury or death [18]. In each of the 12 countries (except Sweden) a study was conducted in at least 30 hospitals depending upon country size and number of hospitals. The selected hospitals represent either all of the relevant institutions in the country (Ireland, Norway) or are randomly selected, per country, from a registry of all general (non-specialized) hospitals. In Belgium, Germany, The Netherlands, Switzerland, England and Spain this selection was done at random within strata (geographical location within the countries, hospital size, and hospital type). In case randomly selected hospitals declined to participate a second or third wave of randomly selected hospitals were invited. In Belgium and Germany, hospitals (that are not selected at random) were also given the opportunity to participate on a voluntary basis. The impact of adding this group of voluntary participating hospitals will be assessed by means of sensitivity analyses. In Finland, Poland and Greece hospitals were selected via purposive sampling (i.e. geographical spread, hospital size, hospital type). Representativeness checks (i.e. hospital type and size) will be carried out in each country to assure the sample represents the population appropriately.

Within each hospital a minimum of 2 nursing units (1 general surgical and 1 general medical nursing unit) were randomly selected from a master list of nursing units. The study sample included only adult medical-surgical care nursing units since the science of linking different elements of nursing practice environment (including nurse staffing) to patient safety and clinical outcomes is best documented within this area [10-13,15,16,19]. Specialized nursing units (e.g. intensive care & high dependency units, transplant care units, pediatric units, geriatric and long-term care nursing units) were excluded from the sampling frame. The minimum number of nursing units per hospital that were sampled varies between the country-specific protocols, ranging from 2 nursing units in Switzerland and Finland to all eligible nursing units in England (with a maximum of 10) and Norway. Six countries (Belgium, The Netherlands, Switzerland, Finland, Spain and Germany) sampled a variable number of nursing units based on hospital size (e.g. Belgium: 4 nursing units in hospitals with <500 beds; 6 nursing units in hospitals with 500 beds or more).

In each country all staff nurses (except nurses on sick leave, maternity leave or those who are on vacation) providing direct care to patients on the selected nursing units were included in the nurse survey. 'Nurses' are defined in each country as those meeting the European Union definition of trained and licensed nurses according to directive 2005/36/EC. In Sweden a different sampling design was used. Nurses were not approached through hospitals but via the Swedish Nursing Association (covering 85% of all nurses). Via the member register all registered nurses employed in hospitals and working in medical and surgical departments were selected. Nurses were asked to identify the hospital in which they work. This method has proved to be effective in previous research [10].

In five countries (Belgium, Poland, Greece, Finland, and Switzerland) all the selected hospitals were included in the patient survey. Whilst in other countries the patient survey was only conducted in a selection (Spain, Germany, and Ireland) or none of the hospitals (The Netherlands, Sweden, and Norway). A one-day census approach was used to select patients of the selected nursing units. All eligible patients (i.e. able to speak and understand the language of the questionnaire and to respond to the questions), present on the selected nursing units on the day of the census, were included in the study sample.

### Instruments & measures

Drawing on previous experience of the 'International Hospital Outcome Study', wherever possible, existing instruments were used [20].

#### Survey general management

The study team in each country collected information about each hospital in their study, including variables about the organizational profile (e.g. size of the hospital in terms of beds and patient activity, the types of technology available, total expenditure), as well as detailed information on staffing for all categories of hospital workers (RNs, second level nurses, unlicensed assistive personnel, physicians and others) and the organization and management of nursing work within the hospital (e.g. methodology used to allocate staff to nursing units). Standard definitions of all of these variables were prepared based on previous experience and expert discussion within the RN4CAST-consortium. Each study team enquired if these data could be drawn from existing databases maintained by governmental or quasi-governmental agencies or if the general management of the selected hospitals had to be questioned to obtain these data. These data will be used to control the analyses for institutional differences.

#### Nurse Survey

Each team conducted surveys of hospital nurses based on a core battery of well-known and extensively validated instruments and questions developed and tested in prior research [10,11]. The survey had two main purposes. The first was to measure, within and across countries, characteristics of the hospital nurse workforce, nurses' future employment intentions, and of nurses' perspectives on quantity and quality of care. The second aim was to allow the creation of hospital- or nursing unit level measures of staffing and working conditions for nurses through aggregation of responses from nurses working in each nursing unit or institution. Previous studies of hospitals in Europe and elsewhere suggest that these properties, which are the result of national workforce policy and local management decisions, influence both the retention of nurses and the quality of patient care [11,20-23]. The survey contains 118 questions comprising nursing work environment, burnout, job satisfaction, nurse-perceived quality of care, nurse staffing levels (number & education), and a demographics section.

The **Practice Environment Scale of the Nursing Work Index or PES-NWI **[24], was used to measure elements of nurses' work environments. The revised PES-NWI consists of 32 Likert type questions (1: "Strongly Disagree" --> 4 "Strongly Agree") including 5 sub-scales: Nurse participation in hospital affairs (8 questions); nursing foundations for quality of care (9 questions); nurse manager ability, leadership and support of nurses (4 questions); staffing and resource adequacy (4 questions); and collegial nurse-physician relationships (7 questions). The reliability (i.e. Cronbach alpha coefficients) of the PES-NWI subscales vary from 0.71 to 0.84 [24]. The subscales have showed to have a high predictive validity for workforce stability issues and quality of care in hospitals [11,25]. The PES-NWI subscales can be combined into a composite measure as either a continuous variable or a three category variable indicating favorable, mixed, or unfavorable practice environments [26].

Burnout, found in front-line human services workers, has important deleterious effects on job satisfaction, nurse turnover, patient satisfaction [27]. The levels of burnout are evaluated by means of the **Maslach Burnout Inventory or MBI **[28]. MBI includes 22 items scored on a scale from 1 "Never" to 6 "Every Day" and is internationally the most widely used instrument for measuring the phenomenon of work-related burnout. MBI captures three dimensions of burnout: emotional exhaustion, depersonalization, personal accomplishment. The three factor structure was largely validated recently in a multi-country study [29].

Rather than using multi-item, comprehensive measures [30] about job satisfaction a single question (with scores ranging from 1 "Very dissatisfied" to 4 "Very satisfied") about overall current **job satisfaction **was employed because of the overlap of existing longer measures with the PES-NWI. Published reliability coefficients for single-item overall job satisfaction are in the range of 0.70-0.80 [31]. In addition, in this study job satisfaction about 9 specific aspects of the job were included in the questionnaire (e.g. Work schedule flexibility, opportunities for advancement, wages).

**Nurse ratings of quality of nursing care **provide related yet distinct information about patient outcomes when compared with statistics derived from hospital discharge databases [32]. The following measures of quality of nursing care from reports on the nurse survey items were created: (1) nurses' reports of the quality of nursing care on their unit, on their last shift, and changes in the quality of nursing care over the last year; (2) readiness of patients for discharge; (3) estimate of the frequency of a variety of adverse events involving themselves and their patients (e.g. medication errors, nosocomial infections, patient falls with injuries, pressure ulcers after admission, urinary tract infection). In addition, 7 questions derived from the AHRQ safety culture questionnaire [33] were included to measure the safety culture in the selected nursing units/hospitals (scoring ranges from 1 " Strongly Disagree" to 5 " Strongly agree").

Difficulties in obtaining consistent **measures of the numbers of nurses working in hospitals **within and across hospitals from administrative or regulatory databases led researchers [10] to develop and refine questionnaire measures of nursing workload/staffing. Each nurse was asked to report, the number of nurses and patients present on the nursing unit and the number of patients cared for during the last shift or workday. Based on these questions nurse-to-patient ratio will be calculated. Nurses were also asked to indicate for their patients the dependence in activities of daily living and need for close monitoring and/or frequent treatments allowing to correct the nurse-to-patient ratio for differences in nursing intensity. The predictive validity of this method of measuring hospital nurses' workloads has been established by the University of Pennsylvania [10,13,34,35].

In the **demographics section **of the questionnaire, specific demographic characteristics of the respondents were gathered for descriptive purposes and these will also be used as explanatory covariates in our modeling including age and sex. Questions were asked regarding the country where each nurse received their basic nursing education, years since first licensure as a nurse, years working in the current country, hospital, and position, and highest achieved level of education in nursing. In research by Aiken et al. [34] and replicated by Estabrooks et al. [36], educational level aggregated to the hospital in terms of the proportion of nurses holding baccalaureate and higher degrees as their highest credential in nursing was found to be predictive of mortality and failure to rescue when aggregated to the hospital.

#### Patient outcomes based on administrative databases

Routinely collected administrative databases, **hospital discharge abstract datasets **in particular, will be used to calculate patient outcomes. Hospital discharge data summarize key information about each hospital stay over a specific time period and contain useful details that can be used to gauge the quality of care being delivered across facilities. Records in these data files include a facility identifier indicating where the hospitalization occurred, patient demographics, characteristics of the admission, principal and secondary International Classification of Diseases (ICD) diagnosis and procedure codes, payer, length of stay, discharge status (alive/dead) and destination, and Diagnosis Related Groups (DRG) assignment. While all participating countries, except Greece, have some type of hospital discharge dataset in use, the specific nomenclatures (ICD-9, ICD-10) and DRG-schemes vary somewhat from country to country as does the history of use and extent to which data have been validated [37]. Despite these differences, the basic coding schemes (such as ICD-9 and ICD-10) are quite similar allowing the use of these data to deduct patient outcome measures in a similar way. Hospital discharge databases will be used to construct hospital-specific patient outcome measures for well-defined types of admissions across hospitals and to analyze these outcomes adjusting for important patient characteristics (such as age, gender and co-morbidities). Principal outcome measures will include risk-adjusted in-hospital mortality and failure-to-rescue, both of which have shown associations with staffing and other nursing-related factors in international research [10,13-15].

#### Patient Survey

Recent research illustrated that higher nurse-to-patient ratios [35,38] and a better nurse work environment [35] are associated with higher levels of patient satisfaction. Both studies used the Consumer Assessment of Healthcare Providers and Systems survey (CAPHS), developed by the Agency for Healthcare Research and Quality [39]. This instrument which asks patients 27 questions about their experiences in the hospital was also used in a slightly shorted form in the RN4CAST study. Three demographic questions were excluded for the EU study: questions about Spanish, Hispanic or Latino origin; questions about race and questions about the language used at home. The items of the survey will be reported as a set of ten measures (six summary measures, two single items, and two global ratings) related to communication with nurses and doctors, responsiveness of hospital staff, pain management, communication about medicines, discharge information, cleanliness and quietness of the hospital environment, overall rating of the hospital, and willingness to recommend the hospital to friends and family.

#### Survey translation

The English core battery of survey instruments was translated into the 10 primary language(s) (Dutch, German, Greek, French, Italian, Finnish, Norwegian, Polish, Swedish, and Spanish) using translation-back translation method. No changes to the template (questions and tools, as well as items within tools) of the core questionnaire were allowed. In each country the quality of the translated instruments were assessed by a panel of 7 to 11 bilingual experts to obtain Content validity indexes for each item separately (I-CVI) and for the entire scale (S-CVI) [40].

#### Data collection procedures

In all of the countries (except Sweden) a field manager was identified in each hospital as key contact with the national RN4CAST team throughout the conduct of the study. In six countries (Norway, England, Spain, Poland, Germany, and Switzerland) the field manager was responsible to distribute questionnaires to the nurses and patients (if applicable) within their hospital whereas in four countries (Belgium, The Netherlands, Ireland, and Greece) the field manager accommodated visits of the research team to the selected nursing units. In the latter group of countries the research team explained the context of the study and distributed the questionnaires. In Finland nurses and patients received the questionnaires via e-mail and the local field manager, respectively. In Sweden nurses received questionnaire packets by mail at their home address. Nurses were asked to return their questionnaires in sealed envelopes in a secured box on the unit (Ireland, Belgium, Netherlands, Spain, Poland, and Greece), via pre-paid envelopes (England, Switzerland, Sweden, and Germany) or online (Finland) within 2-3 weeks. Patients were asked to return the questionnaires by pre-paid envelopes (Spain, Switzerland and Germany) or to hand them to the nurses who can return the questionnaires in sealed envelopes in a secured box on the unit (Belgium, Ireland, Finland, Poland, and Greece). Various approaches were used (post-cards reminders, feedback of response rates to field managers with benchmarks of other hospitals, extra visit of the research team to the nursing unit to re-enforce the teams) to maximize response rates.

The collection of the patient discharge data depends on the nature of the database and the laws and regulations surrounding access of researchers to their use in each country. Different procedures were followed at each site to acquire patient level data about adult hospitalized medical and surgical patients.

### Data analysis

Preliminary analyses of the raw country-specific datasets including descriptive work to identify out-of-range values for variables, conflicting results, missing values and possible data entry errors will be performed. A cleaned version of the different data sources will be organized in an interrelated multilevel meta-database. This multi-country database contains information at the hospital level (survey general management), the individual nurse level (data resulting from the nurse survey) and the patient level (data from the hospital discharge dataset and the patient survey). The cross-country patient outcomes database will not contain all the original data but rather a selection of original variables (e.g. age, sex) and deduced variables (e.g. patient outcomes, diagnostic information, co-morbidities). Patient outcomes and co-morbidities will be based on published work like that of Aiken et al. [10,34], the Charlson Index [41] and the work of Silber et al. [42,43] Cross-mapping will be carried out for the codes used in the different algorithms because the use of different local coding languages and grouping systems (e.g. ICD-9 vs. ICD-10; APR-DRG vs. AP-DRG). This transformation process of internationally available algorithms to the context of local databases has already been done with success by several project partners [13,14].

Two major types of analyses will be done using the multi-country survey and outcomes data. The first will involve descriptive and comparative analyses of variables reflecting commonalities and differences, policy implications and the strengths and weaknesses of the nurse workforce across countries. In these analyses individual level nurse data will be used and aggregated at the country level.

The second type of analysis involves the modeling of relationships between core independent and dependent hospital variables within and across countries. Independent measures will include staffing and work environment variables. Dependent measures will be both indicators of experiences of nurses (e.g. job satisfaction, burnout, intention to leave the job) on the job and patient outcomes variables. Regression models that estimate average differences in continuous outcome variables or differences in the odds of various negative events for nurses and patients will be fitted. The clustering of nurses and patients within hospitals (and, in the case of cross-national analysis, of hospitals within countries) will require the use of multilevel modeling strategies [44]. A five staged approach will be employed. In a first set of analysis the factor structure of the PES-NWI and the risk-adjustment procedures will be explored. Multilevel factor analytic techniques [45] will be used to confirm the structure of the subscales from the PES-NWI to be used as indicators of nurse practice environments. The within- and between-hospital variability of the nursing practice environment components will be studied. This is necessary prior to potential aggregation of some measures to the nursing or hospital level. Logistic regression models will be used with the patient discharge data (patient demographics, co-morbidities, diagnostic categories) to derive propensity scores, based on factors affecting the likelihood of mortality and failure-to-rescue, which will serve as case mix adjusters in further analyses. In a second step, a two-level model (in each country separately) will be applied to study the relationship between hospital characteristics (aggregate measures obtained from the nurse survey such as nursing workload, nursing practice environment) and outcome measures (e.g. nurse assessed quality of care, retention, job satisfaction) obtained at the level of the individual nurse via the survey. In a third step, a two-level model (in each country separately) will be applied to study the relationship between hospital characteristics (aggregate measures obtained from the nurse survey such as nursing workload, nursing practice environment) and patient outcomes (data on the patient level obtained from the hospital discharge datasets or the patient survey) within each country. In a fourth step, the analyses of steps 2 & 3 will be performed across countries thereby introducing a third level (i.e. country: characteristics of the country) into the model allowing cross-country analyses.

Whenever missing data are present, statistical imputation methods will be used to provide alternative analyses (as a sensitivity analysis) to the approaches where cases with missing data are omitted.

### Appraisal of current nurse forecasting models and policy synthesis

An extensive literature review was undertaken to gather information allowing the team to evaluate and appraise the current nurse workforce projection models and forecasts. In addition to searching the traditional databases like CINAHL, Embase and Medline, teams in each country contacted institutions and stakeholders in each country to obtain published and unpublished data and reports about forecasting models. Based on the results of the literature review, the researchers will appraise and evaluate the currently employed forecasting and planning models.

A policy synthesis will combine the literature review on nurse forecasting models with the results of the data analysis about the impact of different aspects of the nursing work environment on patient outcomes and nurses' job experiences. The likely impact of a range of policy instruments (e.g. an increase in nursing education program, investment in recruitment methods, and investments in the nursing practice environment) on patient outcomes and nurse retention and ultimately staffing levels and patient outcomes, will, for instance, be estimated from country-specific data. Sensitivity analyses considering the impact of altering the underlying assumptions in various ways will be used to frame the estimates generated. This will result in several types of country-specific scenarios for the nurse workforce in each country in the coming decades including the simulation of what happens when different conditions in the nursing workforce are changed. A synthesis document will be created and indicating how similar or different the conclusions of the data analyses are across countries, whether or not it is possible to identify clear Europe-wide conclusions from the work in RN4CAST.

### Ethical issues

The project has been granted financial support from the European Commission. Depending on national legislation, the study protocol was approved by either central ethical committees (e.g. nation or university) or local ethical committees (e.g. hospitals). Proof of the ethical approvals has been submitted to the editorial board of this journal for verification. The consortium has developed strict criteria (included in the project proposal and additional internal documents) regarding the sampling of nurses and patients, the storage, flows and access of the data to safeguard the security, privacy and confidentiality.

## Discussion

Human workforce planning in healthcare and patient safety are high on the priority list of international policy organizations. Linking both workforce planning in nursing and patient safety would give a major support to these actions. Nursing is numerically the largest health profession providing direct care. Given their impact on patient outcomes and safety and the costs involved, workforce planning for nursing has significant impacts at a public and policy level [46].

### Policy and scientific impact of the project

The study will make a strong significant scientific contribution by shifting the main focus of nursing workforce planning from rather simple projections in demand and supply of labour to impact on patient safety and quality. The innovative nature of the RN4CAST project is the consideration of new factors in workforce planning such as the work environment recruiting and retention of nurses within the profession and the link between nursing adequacy and patient safety and quality. The study will generate the necessary scientific basis to underpin informed policy decisions on health systems and more effective and efficient strategies of nursing workforce planning. The studies of Needleman et al. [47] and Rothberg et al. [48] show, based on the data collected in the United States, how the research findings within this field of research have become sophisticated enough to be reanalyzed and extrapolated to provide policy guidance. Based on the available data, one proposes that mandating minimum nurse-to-patient ratios in hospitals at 4 or 5 patients per nurse across the United States would yield a cost-benefit ratio (in terms of cost per life saved) superior to that for percutaneous coronary interventions for acute myocardial infarction and routine Pap smears to screen for cervical cancer [48]. The second economic analysis, the extrapolation from results of a major national study of nearly 800 hospitals, suggests that moving all United States general hospitals to a skill mix of highly educated and somewhat more expensive personnel to the 75% percentile nationally would not only save lives, but could actually reduce expenses for the health care system as a whole by lowering complications and shortening length of stay [47]. This latter analysis suggests that the policy approaches increasing skill mix rather than the numbers of personnel would be more effective in improving quality in American hospitals.

Also in Europe, it is illustrated that the potential quality gain in acute hospitals can be substantial. It was shown, for example, that if Belgian acute hospitals would manage to shift the prevalence of "failure-to-rescue" (deaths of patients with complications), to the performance level of the current 20th percentile, the overall in-hospital mortality in Belgian hospitals would decrease with 3.95% in the group of medical patients and with 6.50% in the group of surgical patients [49]. Similar effects were shown in decreasing the number of complications such as hospital acquired infections, pressure sores, wound infections and others.

The study will allow countries to learn from the experience of other health systems and their sustainability, taking into account the importance of national contexts and population characteristics. Focus will be on how nurse staffing and organizational aspects of health systems impact on patient outcomes. The study can serve as an evaluative lens on the nurse workforce impacts of recent national policy initiatives in health care, such as the recent adoption of prospective payment and Diagnostic Related Groups in Germany and the slowing of investments in the National Health Service in England.

### Stakeholders Engagement

Simultaneously to the research activities, the project entails dissemination and stakeholder activities toward achieving the study objectives. An impact assessment preceded the establishment of a stakeholder panel representing patient, nursing and healthcare organizations at the European level. The main role of the international panel is to raise awareness of the project and to support the research team in formulating policy recommendations based on the scientific results. Next to the international stakeholder panel, in each partnering country national stakeholder committees are formed to further gain support for the project.

Next to the large-scale European part of the RN4CAST project, three International Cooperating Partner Countries of the European Union (Botswana, China and South Africa) participate in the project consortium. All three countries will at least perform a pilot study to provide a broader international perspective on the study results.

## List of abbreviations

AHRQ: Agency for Healthcare Research and Quality; CAHPS: Consumer Assessment of Healthcare Providers and Systems; DRG: Diagnosis Related Groups; ICD-9: International Classification of Diseases, 9^th ^edition; ICD-10: International Classification of Diseases, 10^th ^edition; I-CVI: Item Content Validity Index; MBI: Maslach Burnout Inventory; PES-NWI: Practice Environment Scale of the Nursing Work Index; S-CVI: Scale Content Validity Index.

## Competing interests

The authors declare that they have no competing interests.

## Authors' contributions

WS is the principal researcher of the study, LA is co-principal researcher, KV is the day-to-day project and scientific manager. WS, LA and KV equally contributed to developing the research question and study design. WS, LA, KV, AR, PG, MM, RB, RL, AS, LB, TB, JK, MS, LS, DZ equally contributed to implementation of the study protocol. KV drafted the overall manuscript. All those listed as authors were responsible for reading, commenting upon, and approving the final manuscript.

## Pre-publication history

The pre-publication history for this paper can be accessed here:

http://www.biomedcentral.com/1472-6955/10/6/prepub
